# Who may benefit (and who may not) from active ageing? Empirical evidence from European older caregivers, volunteers, and super-helpers

**DOI:** 10.3389/fpubh.2026.1954848

**Published:** 2026-10-07

**Authors:** Federica Cretazzo, Mirko Di Rosa, Andrea Principi

**Affiliations:** 1 Department of Sociology and Social Research, University of Milano-Bicocca, Milan, Italy; 2 Centre for Socio-Economic Research on Ageing, Istituto di Riposo e Cura per Anziani (IRCCS INRCA), Ancona, Italy; 3 Unit of Geriatric Medicine and Clinical Epidemiology, Istituto di Riposo e Cura per Anziani (IRCCS INRCA), Cosenza, Italy

**Keywords:** caregiving, chronic conditions, depressive symptoms, European countries, older adults, self-perceived health, SHARE, super-helping

## Abstract

**Background:**

Against the backdrop of the multidimensionality of the active ageing concept, caregiving and volunteering are recognized as two important domains. Although active ageing is in general considered a preventive tool conducive to positive health outcomes, there is a paucity of studies on health characteristicsoutcomes regarding older adults who are both caregivers and volunteers, by considering their transitions into and from this “double social role”. This is even more so when it comes to studies employing a longitudinal approach. The study investigates, longitudinally, transitions of older Europeans into and from the social role of super-helper (i.e., both caregiver and volunteer) in relation to health-related aspects.

**Methods:**

Relying on data from Waves 7, 8, and 9 of the Survey of Health, Ageing and Retirement in Europe (SHARE), we run regression models to examine the ways changes over time in caregiving, volunteering, and super-helping are associated with chronic conditions, depressive symptoms, and self-perceived health.

**Results:**

The main results evidenced that, transitions from (only) caregiving to super-helping from only caregiving are associated with better health characteristics, while from only volunteering with worse ones. Also, while transitions from super-helping to caregiving are associated with better health characteristics, those ones to volunteering are associated with worse health characteristics. Would be beneficial to co-resident caregivers in terms of fewer depressive symptoms, and that transitions from super-helping into (only) volunteering, in the short-run, may negatively a ffect depressive symptoms (in the case of former co-resident caregivers) and self-perceived health. Volunteering can complement and integrate caregiving through education and training initiatives as well as awareness-raising campaigns, provided that respite care protects older adults against the risks of overburdening. Voluntary organizations should also provide psychological support for older adults who cease caregiving.

## Introduction: background and aims of the study

The European population ageing has been an issue of concern over the last decades. Its causes are well-known among researchers, policy makers, and even in the collective consciousness: increased life expectancy, decreased fertility rates, and low replacement migration (1). In the EU-27, between 2019 and 2050, it is estimated that the number of individuals aged 65–74 will increase by 16.6%, that the number of individuals aged 75–84 will increase by 56.1%, and that the number of individuals aged 85 or over will increase of 113.9% (2). At the same time, in 2050, the dependency ratio—the ratio of individuals aged 65+ to individuals aged 20–64-is expected to be 56.7% (in 2021, it was about 30%). In sum, we are witnessing a rapid population ageing and, what is more important, a rapid rise in the number of the old-old (3). Equally well-known is the fact that this phenomenon has consequences for European economies and welfare systems. Challenges relate to increasing demands for benefits and resources from a growing number of older adults, who may be economically inactive, retired, unemployed, disabled or sick. Regardless of their institutional differences, European welfare systems experience serious difficulties trying to tackle these challenges (4).

Against the backdrop of this phenomenon, the main international organizations and institutions—among which the United Nations Economic Commission for Europe (UNECE), the Organisation for Economic Co-operation and Development (OECD), the European Commission (EC), and also the European Central Bank (ECB)—have promoted the concept of active ageing, which gradually entered the political agenda to help facing these challenges (5). Indeed, population ageing also means that an increasing number of older adults would be able to remain productive and active, if there are favourable policies and accommodating politics. For example, after adopting the EU Pillar of Social Rights (EPSR) in 2017, and after the publication of an action plan on its implementation in 2021, the EU pointed to the relevance of promoting an ageing-friendly environment, where an equitable and high-quality access to services is a social right of every citizen, particularly in older age, and, what is of utmost importance, supports independent living for as long as possible (5). Also, the World Health Organisation (WHO) has proposed a conceptualisation, which involves a paradigm shift regarding ageing (6, 7). Older adults can engage in, or even maintain over time, activities in various spheres of life, for their benefit in terms of health, quality of life, and wellbeing (8). Thus, they can continue to contribute to society and, by doing this, relieving society of public spending and social security burden. In turn, older adults who are engaged in different domains are expected to be healthier and happier (9). A precondition for the latter, is that active ageing should not be a top-down imposition driven by macro-level economic reasons, rather, the way in which to age actively should be freely chosen by older individuals, a choice driven by their interests, motivations, and predispositions (10).

As it has been recognized, two important active ageing domains offering the possibility to fully exercise civil, political, and social rights, so playing a relevant role in society (10, 11), are those of volunteering and caregiving. Both volunteering and caregiving are self-actualizing activities, helping national and local governments to save costs in healthcare, long-term care (LTC), and social care, thus making welfare systems more sustainable. Principi et al. (10) have highlighted the labile line of demarcation between the two activities. While volunteering is a free choice, caregiving may be either free or forced. It could be a forced choice when the caregiver would have avoided performing this task, however cultural norms impose to fulfill care duties and responsibilities, and/or when formal support systems and alternatives are limited (12, 13). In the latter case, caregiving may not fully reflect the active ageing promise of individual positive outcomes. The main aim of this study, is indeed to contribute to understand better the relationship between participation and health, by studying various combinations of transitions in and out of the roles of these two important active ageing domains.

## Prior research

Positive effects of volunteering on individuals in older age are well-known, and the fact that this activity may help European welfare systems in economic and practical terms is evident, since it may compensate for unavailability and unaffordability of formal support services or complement and integrate the existing ones. At the individual level, volunteering may prove to be protective in older age. For example, older adults engaged in volunteering programs have shown to be more likely to initiate a new activity—productive, non-productive, or both—(14) and to adopt successful strategies to face potential challenges that may arise (15). Other benefits of volunteering in older age extend from strengthening self-esteem, shaping personal traits such as compassion and helpfulness, creating opportunities for further reinforcing, or establishing new, informal social networks, to protect individuals’ health and wellbeing (16–18). Using data from the Survey of Health, Ageing, and Retirement in Europe (SHARE) and the Norwegian Life Course, Ageing, and Generation Study (NorLAG), Hansen et al. (16) have found a positive association between volunteering in older age and life satisfaction. Also, relying on SHARE data, Haski-Leventhal (17) has shown that volunteering in older age is positively related to life satisfaction, self-perceived health, and subjective life expectancy. In addition, employing the Health and Retirement Survey (HRS) and the Health and Retirement Survey Consumption and Activities Mail Survey (HRS CAMS), Morrow-Howell et al. (18) have documented that simultaneously engaging in multiple activities in older age is positively associated with self-perceived health, while protecting against depression.

As for caregiving, the fact that this activity is beneficial at the collective level, is quite evident. Informal caregiving may compensate for limited formal support systems, or complement and integrate the existing ones. For instance, as argued in the EC 2009 Ageing Report (19), although LTC public spending—measured as a share of gross domestic product (GDP)—is expected to at least double by 2050, policies and practices promoting caregiving may mitigate this surge. Yet, this does not necessarily translate into LTC cost savings, unless it is framed in the thoughtful design of protective policies and practices supporting caregivers. In fact, it is undeniable that the bulk of care across European countries is shifting beyond formal support, posing the risk of overburdening all informal actors involved (20).

Whether caregiving is beneficial at the individual level is much less clear. A body of literature has focused on the relationship between caregiving and health and wellbeing (21–23). Employing the German Ageing Survey, Zwar et al. (23) have found that transitioning into caregiving, particularly for female and employed individuals, decreases subjective life expectancy, which is both a detrimental factor itself and a predictive factor of health risks. Relying on quantitative data collection from associations, hospitals, parishes, and residencies for older adults, Sánchez-Izquierdo et al. (22) have shown that the perception of a high-quality caregiving relationship increases caregiver’s life satisfaction and quality of life, which may mean that high-quality caregiving is linked with a free choice rather than a forced one. By using the Northern Ireland Mortality Study (NIMS), O’Reilly et al. (21) have documented that caregiving intensity moderates the relationship between caregiving and health. High-intensity caregiving has harmful effects on mental health, whereas low-intensity caregiving has beneficial effects on overall health. Other studies reported negative aspects related to the caregiving activity, in terms of physical and/or mental health, depression, or burden (24, 25). An important aspect, may be whether caregiving is performed inside or outside the household. Related to this, Pelle and colleagues (26) suggested that older caregivers outside the household may hold more resources (i.e., be younger, healthier, more educated, and embedded in a more diversified ego–network of social contacts) than other older people.

What happens when volunteering and caregiving in older age are performed jointly? Notably, the aforecited study of Morrow-Howell et al. (18) has found benefits of simultaneous engagement in multiple activities in older age. They have identified various activity profiles by factoring nine activity domains (such as civic/religious engagement and interpersonal exchange/helping others), using 37 activity items (including volunteering and helping others). Also, Sibalija et al. (27) have shown that being a caregiver improves mental health outcomes, like depression, through the mediating role of other social activities (such as social participation). Other studies have documented the effects of this activity on engagement in other social activities, but nonetheless they have not analysed the implications for health and wellbeing (28, 29).

Adopting an exploratory model based on latent class analysis (LCA) to determine a typology of productive activities and employing data from the American Changing Life (ACL), Burr et al. (20) have found that informal helping and caregiving cluster together, while remaining distinctive activities. This study introduced the concept of *super-helping*, which encompasses individuals engaged in activities motivated by the desire to help others (whether discretionary or obligatory). However, when it comes to volunteering and caregiving, the two activities have been only occasionally jointly studied. One example of this is the aforementioned study by O’Reilly et al. (21), which has documented that, compared to older adults involved in any activity, super-helpers (i.e., older adults involved both in volunteering and caregiving) are less likely to have mobility difficulties.

## Theory and research hypotheses

A body of literature is focusing on the beneficial complementarity of the two activities (and on the multiplicity of any other type of activity) (30–33). Adopting the theory of role extension, which emerges from two foundational works in the field of sociology—the theory of role accumulation (34) and the hypothesis of expansion (35), respectively—this body of literature advocates that the complementarity of volunteering and caregiving may have a positive impact on health and wellbeing. On the contrary, according to theories based on strains and conflicts between multiple roles (36), older individuals may experience tension and burdens due to overlapping social roles. Indeed, there is some empirical evidence on the tension between the roles of volunteering and caregiving (37–40). However, there is currently scarce information about the implications for older adults’ health of volunteering and caregiving (both carried out separately and jointly, i.e., super-helping), while also capturing change over time, as older adults transition between these two social roles. Yet, this could deliver a crucial policy message in order to promote and preserve over time the participation in the two activities. Thus, the questions are: how does health may change over time when older adults transition from participation in a single activity to simultaneous participation in both activities and, conversely, when they shift from engaging in both activities to only one? Do transitions imply positive or negative associations with health-related aspects, over time? Does this pattern hold regardless of whether the initial social role is volunteering or caregiving? These are important questions to be answered in order to better understand whether and in which way to properly promote volunteering and caregiving in older age, as well as the extent to which caregiving may bring individual positive benefits in line with the active ageing concept. This way, not only will welfare systems better tackle the risks posed by the European population ageing, but so too older adults will enjoy better health outcomes.

As argued before, to the best of our knowledge, O’Reilly et al.’s (21) study has been the only one to have shown that super-helping can be positively associated with health-related aspects (i.e., less mobility difficulties). Yet, its cross-sectional design does not allow to observe changes over time, nor does its analytical strategy allow to verify whether this protective effect also applies relative to older adults involved in a single activity. Based on the active ageing underlying assumption of the free choice, as we discussed above, volunteering mostly fulfils this principle, while the same cannot be said for caregiving.

Thus, adopting a longitudinal research design, we assume that the association of volunteering with older adults’ health may be positive in most cases, while the same cannot be true about caregiving. In particular, it is the initial social role that may determine whether a given activity exerts positive or negative associations, providing support in favor of the role extension theory or the role strain theory. The examination of transitions in and out of the two roles, will allow to grasp health-related indications due to change in role configuration, in comparison from health correlates of stable engagement.

In light of this, we hypothesize that:

*Hypothesis 1 (H1)*: Older adults transitioning from caregiving to super-helping (which means caregiving and volunteering) will experience positive associations with health;

*Hypothesis 2 (H2)*: Older adults shifting from super-helping to only caregiving will experience negative associations with health.

On the contrary, we hypothesize that:

*Hypothesis 3 (H3)*: Older adults transitioning from volunteering to super-helping are expected to experience negative associations with health;

*Hypothesis 4 (H4)*: Older adults moving from super-helping to only volunteering are expected to experience positive associations with.

## Materials and methods

### Data

To analyse the time-varying associations of caregiving and volunteering with health conditions of European older adults, we adopt regression analyses using data from Waves 7, 8, and 9 of SHARE (41–57).

SHARE is a large-scale longitudinal sample survey of the European population in older age. Its data collection covers information on different domains of life on a representative sample of individuals aged 50+ living in EU countries, Israel, and Switzerland, and takes place every 2 years, by means of computer-assisted personal interviewing (CAPI) (except for the SHARE Corona Surveys, where computer-assisted telephone interviewing—CATI was the preferred interviewing mode to cope with the social distancing measures related to the COVID-19 pandemic outbreak). SHARE’s ethical approval and informed consent procedures are described in Börsch-Supan and colleagues (47).

Wave 7 fieldwork took place in 2017 (in Portugal, it extended to 2018), administering both a regular panel questionnaire to those respondents who participated in Wave 3 (referred to as SHARELIFE) and a SHARELIFE questionnaire to those respondents who did not participate in Wave 3. We only consider the regular panel interview section. Wave 8 fieldwork started in October 2019 and was interrupted in March 2020, due to the COVID-19 pandemic outbreak, leaving approximately 70% of the sampled individuals issued to the field. For this reason, our base for sample selection at time 0 (t_0_) consists of all cases that are issued to the field at Wave 7, when missing at Wave 8. Since we expect missingness (approximately 30% of the sampled individuals issued to the field) not to be missing-completely-at-random (MCAR), this sample strategy prevented us from smaller sample size, reduced statistical power, and potential bias, even when adopting listwise deletion method. Wave 9 fieldwork lasted between October 2021 and October 2022.

### Sample

For our analytical strategy, we rely on Wave 8 data, augmented with data from Wave 7 where relevant, as the baseline (i.e., t_0_, starting point for transitions between social roles), whereas Wave 9 represents time 1 (t_1_). Also, for time-invariant variables, we feed forward information from previous waves using the last observation carried forwards (LOCF) imputation strategy.

We constructed four analytical samples including Europeans aged 50+ engaged in the different domains under analysis. Subjects who did not participate in Wave 9, were excluded from this study, significantly reducing the sample size. Supplementary Table A1 reports sample characteristics comparing the two groups of individuals included in, and excluded from, the analyses. From this analysis emerged that the two groups were different, in characteristics. Indeed, the excluded individuals were older, had a higher proportion of men, were less educated and more frequently out of the labour market, than the individuals who participated in W9. Three analytical samples consist of older adults involved in caregiving: caregiving outside the household (*N* = 6,733), caregiving inside the household (*N* = 1,667), and caregiving in any context, i.e., regardless of the specific setting (*N* = 8,162). One analytical sample includes those engaged in volunteering (*N* = 4,640). As the way caregiving is conceptualised and measured may impact how we assess transitions between social roles, we replicate statistical analyses considering the types of caregiving and in each of these three cases extend this analytical strategy to volunteering. Tables 1, 2 present the summary statistics.

**Table 1:** Summary statistics regarding older European caregivers.

Variables	%-Means		
Independent variable: caregiving	Caregiving total	Caregiving outside the household	Caregiving inside the household
Only caregiving at t_0_ and t_1_ or super-helping at t_0_ and t_1_ (no change)	81.3	79.6	88.4
Only caregiving at t_0_ and super-helping at t_1_	7.2	8.0	4.1
Super-helping at t_0_ and only caregiving at t_1_	11.4	12.4	7.5

Dependent variables: caregivers’ health conditions						
	Time 0	Time 1	Time 0	Time 1	Time 0	Time 1
Chronic conditions	1.7 (1.6) [0–12]	1.9 (1.7) [0–11]	1.5 (1.5) [0–12]	1.7 (1.6) [0–11]	2.2 (1.9) [0–10]	2.4 (1.9) [0–11]
Depression-EURO-D	2.3 (2.1) [0–12]	2.4 (2.1) [0–12]	2.1 (2.0) [0–11]	2.3 (2.0) [0–12]	3.1 (2.4) [0–12]	3.1 (2.4) [0–12]
Self-perceived health-US version						
Very good or excellent	33.6	30.4	37.4	33.9	18.1	15.6
Good	39.3	40.7	39.9	41.5	36.0	36.8
Poor or fair	27.1	28.9	22.7	24.6	45.9	47.6

Covariates: caregivers’ demographics, socio-economic status, and country						
	Time 0	Time 1	Time 0	Time 1	Time 0	Time 1
Age	66.1 (8.6) [50–99]	68.8 (8.6) [51–102]	64.8 (7.7) [50–98]	67.4 (7.8) [51–100]	72.1 (9.2) [50–99]	74.8 (9.2) [52–102]
Gender						
Male	42.2		44.8		30.0	
Female	57.8		55.2		70.0	
Education						
Low	20.2		15.9		37.7	
Medium	44.6		45.6		41.4	
High	35.2		38.5		20.9	
Current job situation						
Out of the labour market	68.3	75.1	63.6	71.3	88.8	92.2
Economically active	31.7	24.9	36.4	28.7	11.2	7.8
Country	see *footnotes*					

a: Survey of Health, Ageing and Retirement in Europe (SHARE), Waves 7, 8, and 9.

b: Caregiving total: *N* = 8,162. Caregiving outside the household: *N* = 6,733. Caregiving inside the household: *N* = 1,667.

c: %: Percentage. Standard deviation in parentheses. Minimum–maximum in brackets.

d: Countries are: Austria, Belgium, Bulgaria, Croatia, Cyprus, Czech Republic, Denmark, Estonia, Finland, France, Germany, Greece, Hungary, Italy, Israel, Latvia, Lithuania, Luxembourg, Malta, the Netherlands, Poland, Portugal, Romania, Slovakia, Slovenia, Spain, Sweden, and Switzerland.

**Table 2:** Summary statistics regarding older European volunteers.

Variables	%-Means		
Independent variable: volunteering	Caregiving total	Caregiving outside the household	Caregiving inside the household
Only volunteering at t_0_ and t_1_ or super-helping at t_0_ and t_1_ (no change)	61.8	66.6	92.9
Only volunteering at t_0_ and super-helping at t_1_	18.2	14.8	3.9
Super-helping at t_0_ and only volunteering at t_1_	20.0	18.6	3.2

Dependent variables: Volunteers’ health conditions		
	Time 0	Time 1
Chronic conditions	1.6 (1.6) [0–13]	1.7 (1.6) [0–11]
Depression-EURO-D	2.0 (1.8) [0–10]	2.1 (1.9) [0–12]
Self-perceived health-US version		
Very good or excellent	38.0	35.2
Good	40.5	41.4
Poor or fair	21.5	23.4

Covariates: volunteers’ demographics, socio-economic status, and country		
	Time 0	Time 1
Age	67.4 (8.0) [50–99]	70.1 (7.9) [51–101]
Gender		
Male	44.4	
Female	55.6	
Education		
Low	16.5	
Medium	39.4	
High	44.1	
Current job situation		
Out of the labour market	73.9	79.8
Economically active	26.1	20.2
Country	see foototes	

a: Survey of Health, Ageing and Retirement in Europe (SHARE), Waves 7, 8, and 9.

b: Volunteering: *N* = 4640.

c: %: Percentage. Standard deviation in parentheses. Minimum–maximum in brackets.

d: Countries are: Austria, Belgium, Bulgaria, Croatia, Cyprus, Czech Republic, Denmark, Estonia, Finland, France, Germany, Greece, Hungary, Italy, Israel, Latvia, Lithuania, Luxembourg, Malta, the Netherlands, Poland, Portugal, Romania, Slovakia, Slovenia, Spain, Sweden, and Switzerland.

### Dependent variables

Dependent variables are health-related aspects, measured in three ways: (1) the number of chronic conditions; (2) the number of depressive symptoms; and (3) self-perceived health.

For measuring chronic conditions, we created a summary scale using information on 17 health conditions diagnosed by a doctor: heart attack; high blood pressure or hypertension; high blood cholesterol; stroke; diabetes or high blood sugar; chronic lung disease; cancer; stomach or duodenal ulcer, peptic ulcer; Parkinson disease; cataracts; hip fracture or femoral fracture; other fractures; Alzheimer’s disease, dementia, senility; other affective/emotional disorders; rheumatoid arthritis; osteoarthritis/other rheumatism; and chronic kidney disease. Each health condition diagnosed by a doctor or that the respondent reported is a dummy variable. Response categories are: “Yes” and “No.” Since the whole sample answered “No” concerning three conditions, the new measure ranges from 0 to 14, with 0 assigned to individuals who have not been diagnosed with any chronic condition by a doctor.

For measuring depressive symptoms, we used the European Depression Scale (EURO-D). This is a 12-item scale, ranging from 0 to 12, with 0 assigned to those who do not have any depression symptoms.

Self-perceived health is measured with the following question: “Would you say your health is…”. This is a 5-item Likert scale whose response categories are: “Excellent,” “Very good,” “Good,” “Fair,” and “Poor”. The derived variable is coded into three response categories: “Excellent/Very good” (i.e., corresponding to the categories “Excellent” and “Very good”); “Good” (i.e., category “Good”); and “Fair/Poor” (i.e., collapsing the categories “Fair” and “Poor”).

### Independent variables

Caregiving outside the household is measured with the following question: “In the last twelve months, have you personally given any kind of help listed on this card to a family member from outside the household, a friend or neighbour?” Types of help listed in the showcard are: “personal care, e.g. dressing, bathing or showering, eating, getting in or out of bed, using the toilet”, “practical household help, e.g. with home repairs, gardening, transportation, shopping, household chores”, “help with paperwork, such as filling out forms, settling financial or legal matters”. Response categories are: “Yes” and “No”. The derived variable takes value 1 if in the last 12 months the respondent has personally given help and 0 otherwise.

Caregiving inside the household is measured with the following question: “Is there someone living in this household whom you have helped regularly during the last twelve months with personal care, such as washing, getting out of bed, or dressing?” Response categories are: “Yes” and “No.” The derived variable takes value 1 if the respondent has helped regularly during the last 12 months with personal care and 0 otherwise.

We augmented missing values with observed values in the previous waves, if there were observed values on age, caregiving or volunteering. Thus, we managed missingness for those respondents whose age was known and whose engagement in one of the two domains took place at least once. We adopted this sample strategy for caregiving in both settings.

Volunteering is measured with the following question: “Which of the activities listed on this card—if any—have you done in the last twelve months?” The type of activity listed in the card is: “Done voluntary or charity work”. It takes value 1 if the respondent engaged in volunteering or charity work and 0 otherwise. Similar to the sample strategy adopted for caregiving, we augmented missing values with observed values in the previous waves, in the presence of observed values on age, caregiving or volunteering.

We also created a new measure of super-helping, by considering both caregiving and volunteering. We derived a dummy variable taking value 1 if the respondent engaged in both activities and 0 otherwise (i.e., either caregiving or volunteering).

After having created the dummy variables for caregiving, volunteering, and super-helping, we created three variables at baseline: caregiving at t_0_, taking value 1 if the respondent engaged in only caregiving and 0 otherwise; volunteering at t_0_, taking value 1 if the respondent engaged in only volunteering and 0 otherwise; super-helping at t_0_, taking value 1 if the respondent engaged in both activities and 0 otherwise. Similar to the approach adopted for the baseline variables, we created three variables at follow-up: caregiving at t_1_, volunteering at t_1_, and super-helping at t_1_, respectively.

Then, we created new measures for preparing statistical analyses. For caregiving, we derived a categorical variable with three response categories: “Only caregiving at t_0_ and t_1_ or super-helping at t_0_ and t_1_”, “Super-helping at t_0_ and only caregiving at t_1_”, and “Only caregiving at t_0_ and super-helping at t_1_”. For volunteering, we created a similar categorical variable, following the same underlying rationale: “Only volunteering at t_0_ and t_1_ or super-helping at t_0_ and t_1_”, “Super-helping at t_0_ and only volunteering at t_1_”, and “Only volunteering at t_0_ and super-helping at t_1_”. Specifically, the rationale of this was that we wanted to study the effect of transitions between social roles, versus non-transitions/unchanged social roles.

Since these categorical variables include stable trajectories that may differ in health and other characteristics, we ran additional analyses separating stable caregiving, stable volunteering, and stable super-helping into distinct categories (the results of these analyses are provided as Supplementary material). The numbers of participants in each transition category and in different stable groups, are reported in Table 3.

**Table 3:** Participants in transitional and stable roles (*N*).

Caregiving everywhere	*N*
Transitions between social roles	
Only caregiving at time 0 and super-helping at time 1	617
Super-helping at time 0 and only caregiving at time 1	980
Only volunteering at time 0 and super-helping at time 1	565
Super-helping at time 0 and only volunteering at time 1	737
Stable social roles	
Only caregiving at time 0 and time 1	5,407
Only volunteering at time 0 and time 1	1,188
Super-helping at time 0 and time 1	1,665

Caregiving outside the household	*N*
Transitions between social roles	
Only caregiving at time 0 and super-helping at time 1	539
Super-helping at time 0 and only caregiving at time 1	834
Only volunteering at time 0 and super-helping at time 1	650
Super-helping at time 0 and only volunteering at time 1	866
Stable social roles	
Only caregiving at time 0 and time 1	3,873
Only volunteering at time 0 and time 1	1,534
Super-helping at time 0 and time 1	1,504

Caregiving inside the household	*N*
Transitions between social roles	
Only caregiving at time 0 and super-helping at time 1	69
Super-helping at time 0 and only caregiving at time 1	125
Only volunteering at time 0 and super-helping at time 1	175
Super-helping at time 0 and only volunteering at time 1	149
Stable social roles	
Only caregiving at time 0 and time 1	1,318
Only volunteering at time 0 and time 1	3,445
Super-helping at time 0 and time 1	160

### Covariates

As covariates, we considered socio-demographic characteristics, socio-economic status, and country of residence.

Socio-demographic characteristics are age and gender. In the context of demographic demands on the welfare system, not only are those who receive healthcare, LTC, and social care services aging, but so too are those who provide such services (notably, older caregivers). Also, compared to their male counterparts, care duties and responsibilities more heavily fall on women (58). This may impact how transitions between social roles affect health. Age is measured with year of birth in the range of 1916–1970 and computed as years of age at the time of the interview. Gender is measured with a dichotomous variable, taking value 1 if the respondent is a male and 2 if the respondent is a female.

As socio-economic status, educational level and employment status are considered. Having higher educational attainments and being gainfully employed is generally associated with volunteering and simultaneous engagement in the two activities, whereas the opposite is the case observed in relation to caregiving (59). Education is measured with the 1997 International Standard Classification of Education (ISCED) (60). The derived variable has three response categories: “Low” (i.e., corresponding to the categories “None,” “ISCED level 1”, and “ISCED level 2”), “Medium” (i.e., collapsing the categories “ISCED level 3” and “ISCED level 4”), and “High” (i.e., categories “ISCED level 5” and “ISCED level 6”). Employment is measured with the following question: “In general, which of the following best describes your current employment situation?” Response categories are: “Retired”, “Employed or self-employed (including working for family business)”, “Unemployed”, “Permanently sick or disabled”, “Homemaker”, and “Other.” The derived variable is coded into the following response categories: 1 if the respondent is out of the labour market (i.e., corresponding to the categories “Retired”, “Unemployed”, “Permanently sick or disabled”, “Homemaker”, and “Other”) and 2 if the respondent is economically active (i.e., category “Employed or self-employed”).

Country of residence (data not shown) is measured including 26 EU countries—Austria, Belgium, Bulgaria, Croatia, Cyprus, Czech Republic, Denmark, Estonia, Finland, France, Germany, Greece, Hungary, Italy, Latvia, Lithuania, Luxembourg, Malta, the Netherlands, Poland, Portugal, Romania, Slovakia, Slovenia, Spain, Sweden—Switzerland and Israel.

### Analytical strategy

To determine whether changes over time in caregiving, volunteering, and super-helping are associated with the number of chronic conditions and the number of depressive symptoms, we run Poisson regressions. In particular, we run separate statistical models for each of the two active ageing domains (caregiving and volunteering, respectively) and, within each domain, we run four nested (hierarchical) models, meaning that the parameters of one model are a subset of the parameters of the next, which helps when interpreting the results. After having estimated an unadjusted model including only the main independent variable (M1), we add age and gender to verify whether these demographics decide the number of chronic conditions and the number of depressive symptoms (M2). Also, we control for education and employment (M3). In addition, we add country of residence (M4), that is included to control for unobserved, time-invariant, and country-specific effects (also referred to as between-country variability, such as cultural norms).

Furthermore, to determine how changes over time in independent variables may influence self-perceived health, we run ordered logistic regressions, which assumes that the association between each pair of outcome groups is the same (referred to as proportional odds assumption or parallel regression assumption). The proportional-odds assumption was assessed by using the Brant test, confirming the validity of the model. We calculate proportional odds ratios, that we interpret pretty much as we would odds ratios from binary logistic regressions. Similar to the approach adopted for the number of chronic conditions and the number of depressive symptoms, we run separate statistical models for the two active ageing domains. In each of them, we run four nested models: without controls (M1), with socio-demographic characteristics (M2), socio-economic status (M3), and country of residence (M4).

SHARE’s calibrated sampling weights were not applied in the present analysis, as our aim was to examine associations between within-person transitions in social roles and health characteristics, rather than to produce population-representative prevalence estimates. Unweighted regression coefficients provide consistent estimates of such conditional associations, whereas weighting would be required were the goal to estimate population-level prevalence across countries.

Multilevel mixed-effects regressions in M4 were estimated in order to allow total variability to be decomposed into primary level (subject-related variability) and secondary level (country variability).

The most common significance level, i.e., *α* = 0.05, is adopted as the threshold to reject the null hypothesis, which posits that there is no difference between groups or real effect. After estimating models, the likelihood-ratio (LR) test is employed to compare specifications and assess whether the latest specification significantly contributes to the model’s goodness-of-fit. Statistical analyses are all conducted using Stata version 18 (61, 62).

## Results

By concentrating on the transitions focused on the caregiver role, in line with H1, Poisson regression shows that, compared with not experiencing transitions between social roles, transitioning from caregiving to super-helping is significantly associated with a fewer number of chronic conditions after adjusting for socio-demographic characteristics, socio-economic status, and country of residence (M4). Specifically, this association is observed for caregiving total (inside or outside the household) and caregiving outside the household, but not for caregiving inside the household. Also, compared to non-transitioning, transitioning from caregiving to super-helping is significantly associated with a fewer number of depressive symptoms in all the four models This association is only observed for caregiving inside the household.

Similarly, ordered logistic regression shows that, compared to non-transitioning, transitioning from caregiving to super-helping is significantly associated with lower odds of experiencing worse self-perceived health. However, this association is observed for caregiving total after adjusting for socio-demographic characteristics (M2) (OR = 0.834, 95% CI = 0.713–0.976, *p* < 0.05) and for caregiving outside the household in the unadjusted model (M1) (OR = 0.839, 95% CI = 0.712–0.989, *p* < 0.05).

No significant association is observed between transitioning from super-helping to caregiving and chronic conditions, for any type of caregiving, thus not providing empirical evidence for H2. However, contrary to this hypothesis, compared to those who do not experience any transition, those who transition from super-helping to caregiving experience a decrease in the number of depressive symptoms in all the models tested. This association is observed for caregiving total.

In a similar vein, compared to those who do not experience any transition, those who transition from super-helping to caregiving have lower odds ratios of worse self-perceived health. This association is observed for caregiving total, but only in the unadjusted model (M1) (OR = 0.874, 95% CI = 0.767 to 0.987, *p* < 0.05). Table 4 presents the regression results regarding the transitions into and from the caregiver role.

**Table 4:** Chronic conditions, depression, and self-perceived health of older Europeans in light of transitions focused on the caregiver role.

Chronic conditions	M1			M2			M3			M4		
	I	II	III	I	II	III	I	II	III	I	II	III
Super-helping at time 0 and only caregiving at time 1	0.971	1.022	0.931	0.983	1.009	0.964	0.090	1.015	0.975	1.004	1.026	0.977
	(0.025)	(0.029)	(0.058)	(0.025)	(0.028)	(0.060)	(0.026)	(0.028)	(0.061)	(0.026)	(0.029)	(0.062)
Only caregiving at time 0 and super helping at time 1	0.866***	0.881***	0.963	0.911**	0.898**	1.017	0.913**	0.899**	1.014	0.922*	0.911*	1.007
	(0.029)	(0.032)	(0.079)	(0.031)	(0.033)	(0.083)	(0.031)	(0.033)	(0.083)	(0.031)	(0.033)	0.083
Likelihood-ratio test				622.98***	362.31***	64.30***	202.21***	179.90***	25.39***	203.63***	157.06***	34.98***
Pseudo *R*-squared	0.001	0.001	0.000	0.022	0.016	0.010	0.029	0.024	0.014			
Sample size	8,132	6,708	1,662	8,132	6,708	1,662	8,132	6,708	1,662	8,132	6,708	1,662

Depression	M1			M2			M3			M4		
	I	II	III	I	II	III	I	II	III	I	II	III
Super-helping at time 0 and only caregiving at time 1	0.939**	0.980	0.961	0.938**	0.969	0.973	0.948*	0.974	0.991	0.955*	0.976	0.990
	(0.022)	(0.024)	(0.051)	(0.022)	(0.024)	(0.052)	(0.022)	(0.024)	(0.053)	(0.022)	(0.024)	(0.053)
Only caregiving at time 0 and super helping at time 1	0.974	1.037	0.795**	0.995	1.039	0.819*	1.003	1.042	0.823*	1.000	1.038	0.806**
	(0.027)	(0.031)	(0.062)	(0.028)	(0.031)	(0.064)	(0.028)	(0.031)	(0.064)	(0.028)	(0.031)	(0.063)
Likelihood-ratio test				664.64***	460.06***	74.38***	128.51***	64.36***	42.08***	299.09***	191.42***	56.11***
Pseudo *R*-squared	0.000	0.000	0.001	0.019	0.017	0.011	0.023	0.019	0.016			
Sample size	8,132	6,708	1,662	8,132	6,708	1,662	8,132	6,708	1,662	8,132	6,708	1,662

Self-perceived health	M1			M2			M3			M4		
	I	II	III	I	II	III	I	II	III	I	II	III
Super-helping at time 0 and only caregiving at time 1	0.874*	0.982	0.807	0.887	0.969	0.864	0.926	0.994	0.911	0.953	0.997	1.005
	(0.057)	(0.068)	(0.141)	(0.058)	(0.067)	(0.153)	(0.061)	(0.070)	(0.162)	(0.064)	(0.071)	(0.185)
Only caregiving at time 0 and super helping at time 1	0.779**	0.845*	1.129	0.840*	0.864	1.253	0.867	0.878	1.307	0.875	0.874	1.279
	(0.062)	(0.071)	(0.269)	(0.067)	0.073	(0.301)	(0.070)	0.075	(0.316)	(0.073)	0.076	(0.321)
Likelihood-ratio test				281.23***	79.69***	34.38***	301.90***	232.15***	31.72***	675.05***	468.31***	122.67***
Pseudo *R*-squared	0.001	0.000	0.001	0.017	0.006	0.011	0.034	0.022	0.020			
Sample size	8,132	6,708	1,662	8,132	6,708	1,662	8,132	6,708	1,662	8,132	6,708	1,662

a: Survey of Health, Ageing and Retirement in Europe (SHARE), Waves 7, 8, and 9.

b: Reference category = Only caregiving at t_0_ and t_1_ or super-helping at t_0_ and t_1_ for all outcomes.

c: M1 = Model 1 (no adjustments); M2 = Model 2 (added socio-demographic characteristics—age, gender); M3 = Model 3 (added socio-economic status—education, employment); M4 = Model 4 (added country of residence). Standard errors in parentheses. Significance levels: (significant values are shown in bold). *p < 0.05, **p < 0.01, ***p < 0.001.

d: I = Caregiving total; II = Caregiving outside the household; III = Caregiving inside the household.

e: M1, M2, and M3 are estimated using Stata’s *poisson* command for chronic conditions and depression and Stata’s *ologit* command for self-perceived health. M4 is estimated using Stata’s *mepoisson* command for chronic conditions and depression and Stata’s *meologit* command for self-perceived health.

Poisson regressions and ordered logistic regressions. Incidence risk ratios and odds ratios.

As mentioned in the methods, since the reference category combines conceptually different stable trajectories (in this case, individuals who remained caregivers with individuals who remained super-helpers), we replicated the analyses by separating the different stable trajectories (see Supplementary material). While confirming that the two stable trajectories were different, the supplemental analyses confirmed all the results previously obtained augmenting the level of significance, and in some cases these results were reinforced. For instance, concerning H1, significant results obtained in the fully adjusted model in the case of chronic conditions and depressive symptoms, where extended to self-perceived health (in the case of caregivers in total and outside the household).

Concerning H2, the result obtained concerning depressive symptoms was confirmed and extended to self-perceived health (again, significant in the fully adjusted model in the cases of caregiving total and outside the household).

When examining the transitions with a focus on the volunteer role, the data provided empirical evidence for H3, as in the case of the number of depressive symptoms, the negative association with the transition from volunteering to super-helping is significant for co-resident caregivers, and it holds in all the four models tested. Little evidence along these lines was found regarding the number of chronic conditions (unadjusted model).

he results do not provide support for H4 related to chronic conditions, since no significant association is observed between transitioning from super-helping to volunteering and this health outcome, independently from the type of caregiving examined. However, contrary to this hypothesis, compared to non-transitioning, transitioning from super-helping to volunteering is significantly associated with more depressive symptoms when holding all other variables constant (M4). This is observed specifically regarding the transition from caregiving inside the household to volunteering.

Also, compared to older adults who did not experience changes between social roles, those ones who transitioned from super-helping to volunteering have higher odds of experiencing worse self-perceived health. This applies to the sample of caregivers in total (OR = 1.195, 95% CI = 1.038–1.375, *p* < 0.05) and to the sample of caregivers outside the household (OR = 1.193, 95% CI = 1.033–1.376, *p* < 0.05), in both cases when holding all other variables constant (M4). Although a similar path is observed for the sample of caregivers inside the household (OR = 1.386, 95% CI = 1.020–1.883, *p* < 0.05) (M3), in this case the significancyno longer holds after adjusting for the country of residence. Table 5 shows the regression results regarding the transitions into and from the volunteer role.

**Table 5:** Chronic conditions, depression, and self-perceived health of older Europeans in light of transitions focused on the volunteer role.

Chronic conditions	M1			M2			M3			M4		
	I	II	III	I	II	III	I	II	III	I	II	III
Super-helping at time 0 and only volunteering at time 1	1.036	1.025	1.055	1.022	1.010	1.061	1.018	1.009	1.056	1.037	1.027	1.070
	(0.030)	(0.030)	(0.066)	(0.029)	(0.030)	(0.066)	(0.029)	(0.030)	(0.066)	(0.030)	(0.030)	(0.067)
Only volunteering at time 0 and super-helping at time 1	1.034	1.012	1.123*	1.042	1.028	1.103	1.034	1.021	1.091	1.042	1.024	1.103
	(0.031)	(0.033)	(0.063)	(0.032)	(0.034)	(0.061)	(0.031)	(0.034)	(0.061)	(0.032)	(0.034)	(0.062)
Likelihood-ratio test				204.89***	205.07***	204.01***	104.34***	104.70***	104.30***	148.98***	148.29***	148.57***
Pseudo *R*-squared	0.000	0.000	0.000	0.013	0.013	0.013	0.020	0.020	0.020			
Sample size	4,536	4,536	4,536	4,536	4,536	4,536	4,536	4,536	4,536	4,536	4,536	4,536

Depression	M1			M2			M3			M4		
	I	II	III	I	II	III	I	II	III	I	II	III
Super-helping at time 0 and only volunteering at time 1	1.007	1.004	1.180**	1.002	0.998	1.184**	1.001	0.999	1.177**	0.995	0.996	1.166**
	(0.026)	(0.027)	(0.063)	(0.026)	(0.027)	(0.063)	(0.026)	(0.027)	(0.063)	(0.026)	(0.027)	(0.063)
Only volunteering at time 0 and super-helping at time 1	1.027	1.003	1.115*	1.025	0.999	1.121*	1.021	0.995	1.116*	1.024	0.998	1.124*
	(0.028)	(0.030)	(0.056)	(0.028)	(0.030)	(0.057)	(0.028)	(0.030)	(0.057)	(0.028)	(0.030)	(0.057)
Likelihood-ratio test				214.64***	214.75***	215.50***	29.76***	30.03***	29.02***	101.01***	100.71***	100.25***
Pseudo *R*-squared	0.000	0.000	0.001	0.012	0.012	0.013	0.014	0.014	0.014			
Sample size	4,536	4,536	4,536	4,536	4,536	4,536	4,536	4,536	4,536	4,536	4,536	4,536

Self-perceived health	M1			M2			M3			M4		
	I	II	III	I	II	III	I	II	III	I	II	III
Super-helping at time 0 and only volunteering at time 1	1.261**	1.237**	1.406*	1.244**	1.218**	1.423*	1.243**	1.223**	1.389*	1.189*	1.187*	1.312
	(0.088)	(0.088)	(0.219)	(0.087)	(0.087)	(0.222)	(0.088)	(0.088)	(0.217)	(0.085)	(0.087)	(0.209)
Only volunteering at time 0 and super-helping at time 1	1.084	1.062	1.215	1.092	1.081	1.185	1.072	1.062	1.153	1.071	1.060	1.167
	(0.081)	(0.085)	(0.174)	(0.081)	(0.087)	(0.171)	(0.080)	(0.086)	(0.166)	(0.081)	(0.087)	(0.171)
Likelihood-ratio test				46.01***	46.11***	47.10***	100.94***	101.34***	100.18***	249.91***	251.19***	252.17***
Pseudo *R*-squared	0.001	0.001	0.001	0.006	0.006	0.006	0.016	0.016	0.016			
Sample size	4,536	4,536	4,536	4,536	4,536	4,536	4,536	4,536	4,536	4,536	4,536	4,536

a: Survey of Health, Ageing and Retirement in Europe (SHARE), Waves 7, 8, and 9.

b: Reference category = Only volunteering at t_0_ and t_1_ or super-helping at t_0_ and t_1_ for all outcomes.

c: M1 = Model 1 (no adjustments); M2 = Model 2 (added socio-demographic characteristics—age, gender); M3 = Model 3 (added socio-economic status—education, employment); M4 = Model 4 (added country of residence). Standard errors in parentheses. Significance levels: (significant values are shown in bold). *p < 0.05, **p < 0.01, ***p < 0.001.

d: I = Volunteering, when caregiving total; II = Volunteering, when caregiving outside the household; III = Volunteering, when caregiving inside the household.

e: M1, M2, and M3 are estimated using Stata’s *poisson* command for chronic conditions and depression and Stata’s *ologit* command for self-perceived health. M4 is estimated using Stata’s *mepoisson* command for chronic conditions and depression and Stata’s *meologit* command for self-perceived health.

Poisson regressions and ordered logistic regressions. Incidence risk ratios and odds ratios.

Also this case the supplemental analyses (see Supplementary material), still confirming the differences between the two stable trajectories (individuals who remained volunteers and individuals who remained super-helpers), mostly confirmed and reinforced the results. Concerning H3, the result obtained for co-resident caregivers in the case of depressive symptoms, was extended to the sample of caregivers in total. Regarding H4, also, the results obtained in the case of the number of depressive symptoms for caregivers inside the household, were extended to the sample of caregivers in total. As only exception of different results obtained in the supplemental analyses, specifically regarding self-rated health, no significant results in this case were found in the fully adjusted models.

## Discussion

After having entered the political agenda, the active ageing narrative has reflected a very long-lasting and fruitful academic debate, and this also concerns the specific active ageing domains of caregiving and volunteering. However, transitions in time into and from the social roles of caregiver and volunteer, in relation to health, have never been studied, and this was the main reason for carrying out the present study. This paper was aimed to capture the time-varying possible implications of transitions between social roles, compared to stable social roles, for older adults’ health, upon consideration of the type of transition (i.e., from participating in a single activity to simultaneously participating in both activities, super helping, and from engaging in both activities to only one of them), the direction of change over time, and the type of initial social role (i.e., caregiving or volunteering).

Out of the four hypotheses posed, robust results (i.e., statistical significance in the full adjusted models) concerned all the four hypotheses. However, while H1 and H3 were confirmed by the results, in the case of H2 and H4 the results pointed in the opposite direction.

Starting from the first hypothesis, compared to maintaining stable social roles, transitioning from caregiving to super-helping has resulted as being associated with better measures in terms of number of chronic conditions and of depression symptoms, providing empirical evidence in favor of H1, which has been posed according to the role extension theory. Additional analyses (see Supplementary material) extended this result to self-rated health. Applied to the health outcome of chronic conditions (which by definition cannot be improved), this would probably mean that only caregivers with a fewer number of chronic conditions may be able to add, later, the activity of volunteering. Instead, the result that this kind of transition is associated with a fewer number of depressive symptoms among those who were previously only co-resident (with the cared-for relative) caregivers, may suggest that volunteering became a protective factor for this category of caregivers, who are generally those who experience burden and depression due to often round-the-clock and demanding activities, that can negatively affect mental health and depression (21). The same mechanism may apply in the case of self-rated health. H2 hypothesized that, due to the shift from the social role of super-helper to the one of only caregiver, possible health benefits resulting from volunteering would have been lost. Instead, the results provided empirical evidence against this hypothesis in the case of the number of depressive symptoms (and lower odds of worse self-rated health, as tested in the additional analyses). Rather than towards the role extension theory as hypothesized, this result points in the direction of the role strain theory, in which older people with multiple roles may be relieved when giving up one of them. However, this aspect deserves to be investigated more in depth by future research, also in light of the results obtained in this study concerning H4 (see below).

Turning to H3, this hypothesis resulted as supported. The hypothesis was somewhat the opposite of H1. While H1 supposed that adding volunteering to caregiving could have been positive in relation to health, due to documented positive links between volunteering and health (63, 64) and in light of the role extension theory (30–34), H3 supposed that since caregiving may be also a “forced choice” (e.g., in the case no one else in the family can or wants to be involved as caregiver) (65, 66), by adding this activity to volunteering, health could have been negatively associated with the transition mentioned. This is more in line with the view of the role strain theory (36–39) since accumulated and partially undesired social roles may generate tensions and stress. The results of the present study, support this hypothesis in the case of depressive symptoms, while little evidence in their case was found for the number of chronic conditions (significant results only in the unadjusted model).

A further transition under study was the one from super helping to only volunteering. Our hypothesis in this respect (H4) was that, in this case, health implications would have been positive, based on the fact that caregiving could have been a forced choice and maybe undesired social role, and once terminated that kind of activity, volunteer activities would have implied more positive health associations than in the previous situation. However, this hypothesis was not supported, and empirical evidence was found in the opposite direction. Ex caregivers evidenced positive association with higher number of depressive symptoms. In this case, which underlies support for the role extension theory, the motivation for such results may lie in what caused the disengagement from the caregiving activity. Institutionalisation—outsourcing of care duties and responsibilities—or the death of the care receiver are among the main reasons for stopping caregiving (67, 68). Although these events may free up time to invest elsewhere, these former caregivers may not necessarily experience health-related positive associations. Indeed, caregiving relationships often involve affective and emotional bonds and meaningful interactions, based on the quality of the relationship with the care receiver. Once this activity is interrupted, negative feelings, including bereavement-related grief, may emerge and negatively be related to health symptoms.

This study has some limitations, as many factors were not considered in the analyses. For instance, caregiving-related aspects, such as the caregiving intensity and the relationship with the cared-for individual, may moderate the relationship between transitioning between social roles and health (69, 70). Also, the quality of the caregiving relationship and the underlying reasons (i.e., motivations) for transitioning between social roles could shed light on the mechanisms under study. However, due to data partially unavailable in the SHARE sample survey, these variables were not included in the current analyses. Future research should explore whether and in which way these additional aspects interact with transitions between social roles to better understand their relation with health. Also, the study captured transitions between social roles over a short observation window, while patterns over a longer time may differ (e.g., in case of bereavement for the death of an older cared-for relative). However, this short observation window allowed to avoid the potential bias due to multiple measurements of caregiving across all waves of SHARE. As patterns may differ according to the observation window, future research should examine time-varying effects of the transitions between social roles on health by employing other large-scale longitudinal sample surveys of the European population in older age (in addition to SHARE).

Another point is that this study does not control for health at t0, so that it refers to associations rather than to effects. This choice was to avoid the risk of double-counting the same underlying information, due to possible overlap of health at t0 with the outcome definition. Also, conducting multiple statistical tests as in the case of this study, increases the risk of Type I error inflation. Consequently, results should be interpreted with caution and considered as exploratory. The latter, also due to the fact that there were differences in terms of characteristics, between individuals who participated in the study, and those ones who dropped out between t0 and t1.

Against the backdrop of these limitations, the present study adds knowledge concerning the topic of active ageing, since for the first time investigated health in relation to transitions that older European volunteers and caregivers experience into and from the social role of super helper.

## Conclusion

The main results of the present study are that, for older people, adding volunteering to caregiving may be positive in terms of health, while adding caregiving to volunteering may be negative. Also, when super-helpers give up volunteering this may have positive health implications, whereas when they give up caregiving they may experience negative ones. These results partially support the role extension and partially the role strain approaches. This demonstrates that when describing and applying these theories, it is not sufficient to refer to the number of roles, but that the type of roles studied, and the type of transitions in and out of these roles, are fundamental elements.

Relative to the main results, the study delivers important messages for public policy. For example, education and training initiatives as well as awareness-raising campaigns are essential to help individuals and their families understand how volunteering can complement and integrate caregiving with possible positive health-related aspects. This is even more so especially when, once super-helpers, they are supported by public policies providing respite care, which is a crucial long-term service for protecting older adults against the risk of overburdening and allowing them having time and energy for carrying out both activities (caregiving and volunteering). At the same time, it has to be taken into account that that volunteering might be practiced as a respite activity, possibly for short but frequent temporal windows, in light of the results concerning H2. T

Also, for older people, caregiving might be an undesired activity to add to volunteering, given that in this specific case health implications do not seem to be positive. Therefore, this might not reflect the “free choice” option of an activity, which is implicit in the concept of active ageing. The policy level should be conscious that this specific group of older people may need support, related to the caregiving activity. At the same time, the results point in the direction that super-helpers who interrupt the caregiving activity, at least in the short-run, may experience a critical situation in terms of health and depression, so they should be helped through adequate psychological support, that may potentially be organized and offered by means of the volunteer organizations in which they are donating their time and work.

## Data Availability

Publicly available datasets were analyzed in this study. This data can be found here: SHARE Waves 1, 2, 4, 5, 6, 7, 8 and 9 (DOIs: 10.6103/SHARE.w1.900, 10.6103/SHARE.w2.900, 10.6103/SHARE.w4.900, 10.6103/SHARE.w5.900, 10.6103/SHARE.w6.900, 10.6103/SHARE.w7.900, 10.6103/SHARE.w8.900, 10.6103/SHARE.w9.900) [see (47) for methodological details].
