# Supplementary material for: Who may benefit (and who may not) from active ageing? Empirical evidence from European older caregivers, volunteers, and super-helpers
Source: Front Public Health. 2026 Oct 7;14:1954848. doi: 10.3389/fpubh.2026.1954848 (PMC13684909; doi:10.3389/fpubh.2026.1954848)
Supplement: Supplementary file 1 [file Table_1.docx]

**Supplementary material**

**Table A1: Differences in characteristics of dropped-out subjects in Wave 9**

|  | **Total sample** | **Included** | **Excluded** | **p-value** |
| --- | --- | --- | --- | --- |
|  | N = 88,999 | N = 54,052 | N = 34,947 |  |
| Age, Mean (Std. Dev.) | 69.3 (10.3) | 68.4(9.4) | 70.7 (11.3) | <0.001 |
| Gender**,** N (%) |  |  |  | <0.001 |
| Male | 38,504 (43.3) | 22,603 (41.8) | 15,901 (45.5) |  |
| Female | 50, 495(56.7) | 31,449 (58.2) | 19,046 (54.5) |  |
| Education, N (%) |  |  |  | <0.001 |
| Low | 31,144 (35.0) | 17,140 (31.7) | 14,004 (40.1) |  |
| Medium | 37,623 (42.3) | 23,845 (44.1) | 13,778 (39.4) |  |
| High | 20,232 (22.7) | 13,067 (24.2) | 7,165 (20.5) |  |
| Current job situation, N (%) |  |  |  | <0.001 |
| Out of the labour market | 69,055 (77.6) | 41,464 (76.7) | 27,591 (79.0) |  |
| Economically active | 19,944 (22.4) | 12, 588 (23.3) | 7,356 (21.0) |  |

*Abbreviations:* Std. Dev. = Standard Deviation. % = Percentage. N = Number.

**Table A2. Chronic conditions, depression, and self-perceived health of older Europeans in light of transitions focused on the caregiver role. Poisson regressions and ordered logistic regressions. Incidence risk ratios and odds ratios**

| *Chronic conditions* | M1 | | | M2 | | | M3 | | | M4 | | |
| --- | --- | --- | --- | --- | --- | --- | --- | --- | --- | --- | --- | --- |
|  | I | II | III | I | II | III | I | II | III | I | II | III |
| Super helping at time 0 and super helping at time 1 | **0.855***** | **0.908***** | 0.904 | **0.879***** | **0.892***** | 0.948 | **0.890***** | **0.902***** | 0.956 | **0.923**** | **0.927**** | 1.004 |
|  | (0.019) | (0.021) | (0.051) | (0.019) | (0.021) | (0.054) | (0.020) | (0.021) | (0.055) | (0.021) | (0.023) | (0.060) |
| Super helping at time 0 and only caregiving at time 1 | **0.937*** | 0.995 | 0.921 | 0.955 | 0.978 | 0.958 | 0.963 | 0.986 | 0.970 | 0.984 | 1.004 | 0.977 |
|  | (0.025) | (0.029) | (0.058) | (0.025) | (0.028) | (0.060) | (0.025) | (0.028) | (0.061) | (0.026) | (0.029) | (0.062) |
| Only caregiving at time 0 and super helping at time 1 | **0.836***** | **0.858***** | 0.953 | **0.884***** | **0.870***** | 1.011 | **0.888***** | **0.874***** | 1.009 | **0.904**** | **0.892**** | 1.007 |
|  | (0.028) | (0.032) | (0.078) | (0.030) | (0.032) | (0.083) | (0.030) | (0.032) | (0.083) | (0.031) | (0.033) | (0.084) |
| Likelihood-ratio test |  |  |  | 605.49*** | 369.32*** | 61.94*** | 195.00*** | 174.78*** | 25.09*** | 187.59*** | 147.57*** | 34.38*** |
| Pseudo R-squared | 0.003 | 0.001 | 0.001 | 0.023 | 0.017 | 0.010 | 0.030 | 0.024 | 0.014 |  |  |  |
| Sample size | 8,132 | 6,708 | 1,662 | 8,132 | 6,708 | 1,662 | 8,132 | 6,708 | 1,662 | 8,132 | 6,708 | 1,662 |
| *Depression* | M1 | | | M2 | | | M3 | | | M4 | | |
|  | I | II | III | I | II | III | I | II | III | I | II | III |
| Super helping at time 0 and super helping at time 1 | **0.865***** | **0.922***** | 0.910 | **0.885***** | **0.931**** | 0.929 | **0.905***** | **0.944**** | 0.952 | **0.920***** | **0.943**** | 0.995 |
|  | (0.016) | (0.019) | (0.045) | (0.017) | (0.019) | (0.046) | (0.017) | (0.020) | (0.047) | (0.018) | (0.020) | (0.052) |
| Super helping at time 0 and only caregiving at time 1 | **0.908***** | 0.958 | 0.951 | **0.912***** | **0.951*** | 0.965 | **0.926**** | 0.959 | 0.985 | **0.935**** | 0.960 | 0.989 |
|  | (0.021) | (0.024) | (0.051) | (0.021) | (0.024) | (0.052) | (0.022) | (0.025) | (0.053) | (0.022) | (0.025) | (0.054) |
| Only caregiving at time 0 and super helping at time 1 | **0.942*** | 1.014 | **0.787**** | 0.967 | 1.019 | **0.813**** | 0.979 | 1.026 | **0.818*** | 0.980 | 1.020 | **0.806**** |
|  | (0.026) | (0.030) | (0.061) | (0.027) | (0.031) | (0.063) | (0.028) | (0.031) | (0.064) | 0.028 | (0.031) | (0.064) |
| Likelihood-ratio test |  |  |  | 646.60*** | 456.15*** | 72.87*** | 113.67*** | 60.11*** | 40.83*** | 289.67*** | 191.30*** | 55.12*** |
| Pseudo R-squared | 0.002 | 0.001 | 0.002 | 0.021 | 0.017 | 0.011 | 0.024 | 0.019 | 0.016 |  |  |  |
| Sample size | 8,132 | 6,708 | 1,662 | 8,132 | 6,708 | 1,662 | 8,132 | 6,708 | 1,662 | 8,132 | 6,708 | 1,662 |
| *Self-perceived health* | M1 | | | M2 | | | M3 | | | M4 | | |
|  | I | II | III | I | II | III | I | II | III | I | II | III |
| Super helping at time 0 and super helping at time 1 | **0.538***** | **0.644***** | **0.509***** | **0.549***** | **0.626***** | **0.553***** | **0.597***** | **0.666***** | **0.608**** | **0.663***** | **0.707***** | 0.755 |
|  | (0.029) | (0.037) | (0.080) | (0.030) | (0.036) | (0.087) | (0.033) | (0.038) | (0.097) | (0.038) | (0.043) | (0.130) |
| Super helping at time 0 and only caregiving at time 1 | **0.751***** | **0.868*** | 0.748 | **0.767***** | **0.850*** | 0.805 | **0.814**** | 0.887 | 0.855 | **0.854*** | 0.899 | 0.963 |
|  | (0.050) | (0.062) | (0.132) | (0.051) | (0.061) | (0.143) | (0.055) | (0.064) | (0.153) | (0.059) | (0.066) | (0.179) |
| Only caregiving at time 0 and super helping at time 1 | **0.669***** | **0.747**** | 1.048 | **0.725***** | **0.758**** | 1.167 | **0.760**** | **0.783**** | 1.224 | **0.783**** | **0.789**** | 1.232 |
|  | (0.054) | (0.064) | (0.250) | (0.059) | (0.065) | (0.281) | (0.062) | (0.068) | (0.297) | (0.066) | (0.070) | (0.310) |
| Likelihood-ratio test |  |  |  | 270.31*** | 87.18*** | 29.79*** | 266.87*** | 213.97*** | 27.36*** | 636.18*** | 451.37*** | 115.73*** |
| Pseudo R-squared | 0.008 | 0.005 | 0.006 | 0.024 | 0.011 | 0.015 | 0.039 | 0.025 | 0.023 |  |  |  |
| Sample size | 8,132 | 6,708 | 1,662 | 8,132 | 6,708 | 1,662 | 8,132 | 6,708 | 1,662 | 8,132 | 6,708 | 1,662 |

a: Survey of Health, Ageing and Retirement in Europe (SHARE), Waves 7, 8, and 9.

b: Reference category = Only caregiving at t_0_ and t_1_ for all outcomes.

c: M1 = Model 1 (no adjustments); M2 = Model 2 (added socio-demographic characteristics - age, gender); M3 = Model 3 (added socio-economic status - education, employment); M4 = Model 4 (added country of residence). Standard errors in parentheses. Significance levels (significant values are shown in bold): * p < 0.05, ** p < 0.01, *** p < 0.001.

d: I = Caregiving total; II = Caregiving outside the household; III = Caregiving inside the household.

e: M1, M2, and M3 are estimated using Stata’s *poisson* command for chronic conditions and depression and Stata's *ologit* command for self-perceived health. M4 is estimated using Stata’s *mepoisson* command for chronic conditions and depression and Stata’s *meologit* command for self-perceived health.

**Table A3. Chronic conditions, depression, and self-perceived health of older Europeans in light of transitions focused on the volunteer role. Poisson regressions and ordered logistic regressions. Incidence risk ratios and odds ratios**

| *^Chronic conditions^* | ^M1^ | | | ^M2^ | | | ^M3^ | | | ^M4^ | | |
| --- | --- | --- | --- | --- | --- | --- | --- | --- | --- | --- | --- | --- |
|  | ^I^ | ^II^ | ^III^ | ^I^ | ^II^ | ^III^ | ^I^ | ^II^ | ^III^ | ^I^ | ^II^ | ^III^ |
| ^Super helping at time 0 and super helping at time 1^ | **^0.928*^** | **^0.915**^** | **^1.295***^** | ^1.008^ | ^0.996^ | **^1.233***^** | ^1.010^ | ^0.997^ | **^1.212**^** | ^1.028^ | ^1.014^ | **^1.207**^** |
|  | ^(0.027)^ | ^(0.025)^ | ^(0.071)^ | ^(0.030)^ | ^(0.028)^ | ^(0.068)^ | ^(0.030)^ | ^(0.028)^ | ^(0.067)^ | ^(0.031)^ | ^(0.030)^ | ^(0.067)^ |
| ^Super helping at time 0 and only volunteering at time 1^ | ^0.998^ | ^0.982^ | ^1.067^ | ^1.026^ | ^1.001^ | ^1.071^ | ^1.023^ | ^1.007^ | ^1.065^ | ^1.051^ | ^1.034^ | ^1.080^ |
|  | ^(0.032)^ | ^(0.032)^ | ^(0.067)^ | ^(0.033)^ | ^(0.032)^ | ^(0.067)^ | ^(0.033)^ | ^(0.032)^ | ^(0.066)^ | ^(0.034)^ | ^(0.034)^ | ^(0.068)^ |
| ^Only volunteering at time 0 and super helping at time 1^ | ^0.996^ | ^0.969^ | **^1.136*^** | ^1.046^ | ^1.026^ | ^1.113^ | ^1.038^ | ^1.019^ | ^1.101^ | ^1.056^ | ^1.031^ | ^1.113^ |
|  | ^(0.033)^ | ^(0.034)^ | ^(0.063)^ | ^(0.035)^ | ^(0.037)^ | ^(0.062)^ | ^(0.035)^ | ^(0.036)^ | ^(0.061)^ | ^(0.036)^ | ^(0.037)^ | ^(0.062)^ |
| ^Likelihood-ratio test^ |  |  |  | ^198.29***^ | ^194.87***^ | ^197.07***^ | ^104.38***^ | ^104.68***^ | ^102.20***^ | ^149.68***^ | ^148.52***^ | ^148.11***^ |
| ^Pseudo R-squared^ | ^0.001^ | ^0.001^ | ^0.002^ | ^0.013^ | ^0.013^ | ^0.014^ | ^0.020^ | ^0.020^ | ^0.021^ |  |  |  |
| ^Sample size^ | ^4,536^ | ^4,536^ | ^4,536^ | ^4,536^ | ^4,536^ | ^4,536^ | ^4,536^ | ^4,536^ | ^4,536^ | ^4,536^ | ^4,536^ | ^4,536^ |
| *^Depression^* | ^M1^ | | | ^M2^ | | | ^M3^ | | | ^M4^ | | |
|  | ^I^ | ^II^ | ^III^ | ^I^ | ^II^ | ^III^ | ^I^ | ^II^ | ^III^ | ^I^ | ^II^ | ^III^ |
| ^Super helping at time 0 and super helping at time 1^ | **^1.091**^** | **^1.056*^** | **^1.430***^** | **^1.114***^** | **^1.079**^** | **^1.376***^** | **^1.118***^** | **^1.084**^** | **^1.363***^** | **^1.137***^** | **^1.099***^** | **^1.355***^** |
|  | ^(0.029)^ | ^(0.027)^ | ^(0.068)^ | ^(0.030)^ | ^(0.28)^ | ^(0.066)^ | ^(0.030)^ | ^(0.028)^ | ^(0.065)^ | ^(0.031)^ | ^(0.029)^ | ^(0.065)^ |
| ^Super helping at time 0 and only volunteering at time 1^ | ^1.054^ | ^1.032^ | **^1.199**^** | ^1.058^ | ^1.036^ | **^1.201**^** | ^1.060^ | ^1.039^ | **^1.194**^** | **^1.062*^** | ^1.043^ | **^1.184**^** |
|  | ^(0.031)^ | ^(0.030)^ | ^(0.064)^ | ^(0.031)^ | ^(0.031)^ | ^(0.064)^ | ^(0.031)^ | ^(0.031)^ | ^(0.064)^ | ^(0.032)^ | ^(0.031)^ | ^(0.064)^ |
| ^Only volunteering at time 0 and super helping at time 1^ | **^1.075*^** | ^1.031^ | **^1.133*^** | **^1.084**^** | ^1.038^ | **^1.138*^** | **^1.082*^** | ^1.037^ | **^1.132*^** | **^1.094**^** | ^1.047^ | **^1.141*^** |
|  | ^(0.033)^ | ^(0.033)^ | ^(0.057)^ | ^(0.033)^ | ^(0.034)^ | ^(0.058)^ | ^(0.033)^ | ^(0.034)^ | ^(0.057)^ | ^(0.034)^ | ^(0.034)^ | ^(0.058)^ |
| ^Likelihood-ratio test^ |  |  |  | ^219.69***^ | ^218.67***^ | ^205.46***^ | ^30.87***^ | ^31.20***^ | ^26.72***^ | ^105.93***^ | ^103.91***^ | ^98.87***^ |
| ^Pseudo R-squared^ | ^0.001^ | ^0.000^ | ^0.004^ | ^0.013^ | ^0.013^ | ^0.015^ | ^0.015^ | ^0.014^ | ^0.017^ |  |  |  |
| ^Sample size^ | ^4,536^ | ^4,536^ | ^4,536^ | ^4,536^ | ^4,536^ | ^4,536^ | ^4,536^ | ^4,536^ | ^4,536^ | ^4,536^ | ^4,536^ | ^4,536^ |
| *^Self-perceived health^* | ^M1^ | | | ^M2^ | | | ^M3^ | | | ^M4^ | | |
|  | ^I^ | ^II^ | ^III^ | ^I^ | ^II^ | ^III^ | ^I^ | ^II^ | ^III^ | ^I^ | ^II^ | ^III^ |
| ^Super helping at time 0 and super helping at time 1^ | **^0.707***^** | **^0.681***^** | **^1.720***^** | **^0.767***^** | **^0.738***^** | **^1.628**^** | **^0.776***^** | **^0.752***^** | **^1.557**^** | ^0.872^ | **^0.844*^** | **^1.557**^** |
|  | ^(0.050)^ | ^(0.046)^ | ^(0.258)^ | ^(0.055)^ | ^(0.051)^ | ^(0.245)^ | ^(0.056)^ | ^(0.052)^ | **^(0.236)^** | ^(0.065)^ | ^(0.061)^ | ^(0.239)^ |
| ^Super helping at time 0 and only volunteering at time 1^ | ^1.058^ | ^1.023^ | **^1.435*^** | ^1.089^ | ^1.051^ | **^1.449*^** | ^1.094^ | ^1.065^ | **^1.413*^** | ^1.110^ | ^1.093^ | ^1.335^ |
|  | ^(0.083)^ | ^(0.081)^ | ^(0.224)^ | ^(0.086)^ | ^(0.083)^ | ^(0.226)^ | ^(0.087)^ | ^(0.084)^ | ^(0.221)^ | ^(0.090)^ | ^(0.089)^ | ^(0.213)^ |
| ^Only volunteering at time 0 and super helping at time 1^ | ^0.909^ | ^0.878^ | ^1.241^ | ^0.954^ | ^0.928^ | ^1.207^ | ^0.942^ | ^0.921^ | ^1.173^ | ^0.997^ | ^0.974^ | ^1.188^ |
|  | ^(0.075)^ | ^(0.076)^ | ^(0.178)^ | ^(0.079)^ | ^(0.081)^ | ^(0.174)^ | ^(0.078)^ | ^(0.081)^ | ^(0.169)^ | ^(0.085)^ | ^(0.087)^ | ^(0.174)^ |
| ^Likelihood-ratio test^ |  |  |  | ^35.39***^ | ^33.14***^ | ^44.45***^ | ^99.71***^ | ^98.85***^ | ^98.27***^ | ^240.91***^ | ^239.96***^ | ^251.94***^ |
| ^Pseudo R-squared^ | ^0.004^ | ^0.004^ | ^0.002^ | ^0.007^ | ^0.008^ | ^0.007^ | ^0.018^ | ^0.018^ | ^0.017^ |  |  |  |
| ^Sample size^ | ^4,536^ | ^4,536^ | ^4,536^ | ^4,536^ | ^4,536^ | ^4,536^ | ^4,536^ | ^4,536^ | ^4,536^ | ^4,536^ | ^4,536^ | ^4,536^ |

a: Survey of Health, Ageing and Retirement in Europe (SHARE), Waves 7, 8, and 9.

b: Reference category = Only volunteering at t_0_ and t_1_ for all outcomes.

c: M1 = Model 1 (no adjustments); M2 = Model 2 (added socio-demographic characteristics - age, gender); M3 = Model 3 (added socio-economic status - education, employment); M4 = Model 4 (added country of residence). Standard errors in parentheses. Significance levels (significant values are shown in bold): * p < 0.05, ** p < 0.01, *** p < 0.001.

d: I = Volunteering, when caregiving total; II = Volunteering, when caregiving outside the household; III = Volunteering, when caregiving inside the household.

e: M1, M2, and M3 are estimated using Stata’s *poisson* command for chronic conditions and depression and Stata's *ologit* command for self-perceived health. M4 is estimated using Stata’s *mepoisson* command for chronic conditions and depression and Stata’s *meologit* command for self-perceived health.
